# Utilization of L-glutamate as a preferred or sole nutrient in *Pseudomonas aeruginosa* PAO1 depends on genes encoding for the enhancer-binding protein AauR, the sigma factor RpoN and the transporter complex AatJQMP

**DOI:** 10.1186/s12866-021-02145-x

**Published:** 2021-03-15

**Authors:** Benjamin R. Lundgren, Joseph M. Shoytush, Ryan A. Scheel, Safreen Sain, Zaara Sarwar, Christopher T. Nomura

**Affiliations:** 1 Department of Chemistry, State University of New York - College of Environmental Science and Forestry, Syracuse, 1 Forestry Drive, Syracuse, New York, 13210 USA; 2 Department of Biology, The College of New Jersey, 2000 Pennington Road, Ewing, NJ 08628 USA; 3 Department of Biological Sciences, University of Idaho, 875 Perimeter Drive, Moscow, ID 83844 USA

**Keywords:** Enhancer-binding protein, Acidic amino acids, Glutamate utilization, AauR, RpoN, *Pseudomonas aeruginosa*

## Abstract

**Background:**

Glutamate and aspartate are preferred nutrients for a variety of microorganisms. In the case for many *Pseudomonas* spp., utilization of these amino acids is believed to be dependent on a transporter complex comprised of a periplasmic-solute binding protein (AatJ), two permease domains (AatQM) and an ATP-binding component (AatP). Notably, expression of this transporter complex is hypothesized to be regulated at the transcriptional level by the enhancer-binding protein AauR and the alternative sigma factor RpoN. The purpose of the current study was to determine the biological significance of the putative *aatJ-aatQMP* operon and its regulatory *aauR* and *rpoN* genes in the utilization of L-glutamate, L-glutamine, L-aspartate and L-asparagine in *Pseudomonas aeruginosa* PAO1.

**Results:**

Deletion of the *aatJ-aatQMP*, *aauR* or *rpoN* genes did not affect the growth of *P. aeruginosa* PAO1 on L-glutamate, L-glutamine, L-aspartate and L-asparagine equally. Instead, only growth on L-glutamate as the sole carbon source was abolished with the deletion of any one of these genes. Interestingly, growth of the *aauR* mutant on L-glutamate was readily restored via plasmid-based expression of the *aatQMP* genes, suggesting that it is the function of AatQMP (and not AatJ) that is limiting in the absence of the *aauR* gene. Subsequent analysis of beta-galactosidase reporters revealed that both *aatJ* and *aatQ* were induced in response to L-glutamate, L-glutamine, L-aspartate or L-asparagine in a manner dependent on the *aauR* and *rpoN* genes. In addition, both *aatJ* and *aatQ* were expressed at reduced levels in the absence of the inducing-amino acids and the regulatory *aauR* and *rpoN* genes. The expression of the *aatJ*-*aatQMP* genes is, therefore, multifaceted. Lastly, the expression levels of *aatJ* were significantly higher (> 5 fold) than that of *aatQ* under all tested conditions.

**Conclusions:**

The primary function of AauR in *P. aeruginosa* PAO1 is to activate expression of the *aatJ-aatQMP* genes in response to exogenous acidic amino acids and their amide derivatives. Importantly, it is the AauR-RpoN mediated induction of the *aatQMP* genes that is the pivotal factor enabling *P. aeruginosa* PAO1 to effectively utilize or consume L-glutamate as a sole or preferred nutrient.

## Background

The assimilation or utilization of dicarboxylates from the environment is wide-spread among soil-dwelling and plant-associated bacteria. For some bacteria, dicarboxylates serve as preferred nutrients for cellular growth whereas for others consumption of these compounds is an important component of their complicated lifestyles. An example of the latter is the establishment of symbiotic relationships between *Rhizobia* and plants; the exudates from plants are a rich source of C_4_-dicarobxylates, especially malate [1, 2]. Bacteria belonging to the genus *Pseudomonas* are some of the best-known examples of the former in which both C_4_- and C_5_-dicarboxylates are at the top of the hierarchy for carbon source utilization [3, 4].

In the two instances given above it is the transport of dicarboxylates across the bacterial membrane that is often a major control point or regulated step in their utilization. Regulation of dicarboxylate transport is commonly achieved through a two-component signal transduction system (TCS) comprised of the histidine sensor kinase DctB and its cognate response regulator DctD [1, 5]. In the typical DctBD TCS, extracellular dicarboxylates are expected to bind to the periplasmic input domain of DctB and stimulate the phosphorylation of a conserved histidine residue within its cytoplasmic transmitter domain. The phosphoryl group is subsequently transferred from the phosphohistidine of DctB to a conserved aspartate residue located in the response-regulator domain of DctD. Phosphorylated DctD then activates or upregulates the transcription of genes encoding for dicarboxylate-related transport proteins. In addition to being a response regulator, DctD is part of an unusual family of bacterial transcriptional regulators called enhancer-binding proteins or EBPs [6, 7]. The unusual or unique functionality of EBPs is due to the fact that they are the only known family of transcriptional regulators that activate transcription from the − 12/− 24 promoters recognized by the alternative sigma factor RpoN [8–10]. In other words, transcription from RpoN-controlled − 12/− 24 promoters are dependent on EBPs. This means that genes under the regulation of the DctBD TCS are also potentially governed by RpoN in the bacterial cell.

Interestingly, the genomes of various *Pseudomonas* spp. encode for three distinct DctBD TCSs wherein each one is specific towards a particular class of dicarboxylates. Two of these systems have been characterized for *Pseudomonas aeruginosa* PAO1. The classical or prototypical DctBD TCS of *P. aeruginosa* PAO1 is encoded by the *PA5165* – *PA5166* genes, and regulates the transport of C_4_-dicarboxylates, including succinate, fumarate and malate [11]. Specifically, DctD activates transcription of genes encoding for a C_4_-dicarboxylate-sodium symporter known as DctA and a tripartite ATP-independent periplasmic or TRAP transporter called DctPQM. The second characterized DctBD TCS of *P. aeruginosa* consists of the sensor kinase MifS (PA5512) and its cognate response regulator MifR (PA5511). The MifSR TCS was originally identified for its role in microcolony formation and named accordingly [12]. It was later demonstrated that the MifSR TCS is required for the utilization of C_5_-dicarboxylates such as α-ketoglutarate (α-KG) in which MifR activates transcription of the *PA5530* gene, encoding for a C_5_-dicarboxylate-proton symporter [13–15]. The utilization of C_4_- and C_5_-dicarboxylates as a sole carbon source in *P. aeruginosa* PAO1 is dependent on DctD and MifR, respectively [11, 13].

The third and final DctBD TCS occurring in *P. aeruginosa* PAO1 is that of the acidic-amino acid utilization regulator AauR (PA1335) and its partner sensor histidine kinase AauS (PA1336). The AauSR TCS has only been investigated in *Pseudomonas putida* KT2440 where it was shown to have an influential effect in the utilization of L-glutamate, L-glutamine and L-aspartate [16, 17]. Notably, expression of an ATP-binding cassette (ABC) transporter complex encoded by the *aatJ*-*aatQMP* genes was observed to be upregulated in response to L-glutamate, and the *aauR* gene was implicated as being required for full expression of this gene cluster in *P. putida* KT2440 [16]. Subsequent analysis revealed that the periplasmic solute-binding protein AatJ exhibits affinity constants of ~ 0.3 and 1.5 μM for L-glutamate and L-aspartate, respectively, while deletion of the *aatJ*-*aatQMP* locus in *P. putida* KT2440 resulted in drastic growth reductions on L-glutamate, L-aspartate or L-glutamine if present as the sole source of carbon and nitrogen [17]. DNase I footprinting experiments suggest that AauR binds to the consensus sequence TTCGG-N_4_-CCGAA located in the 5′-regulatory or promoter region of the *aatJ*-*aatQMP* operon [17]. Putative AauR-binding sites were also found within the 5′-regulatory regions of *gltP* (glutamate/aspartate symporter), *ppsA* (phosphoenolpyruvate synthase), *ansB* (glutaminase/asparaginase) and *dsbC* (thiol/disulfide exchange protein), arguing that the AauSR TCS regulates a consortium of genes in response to L-glutamate, L-glutamine and/or L-aspartate in *P. putida* KT2440 [17].

**Fig. 1:**
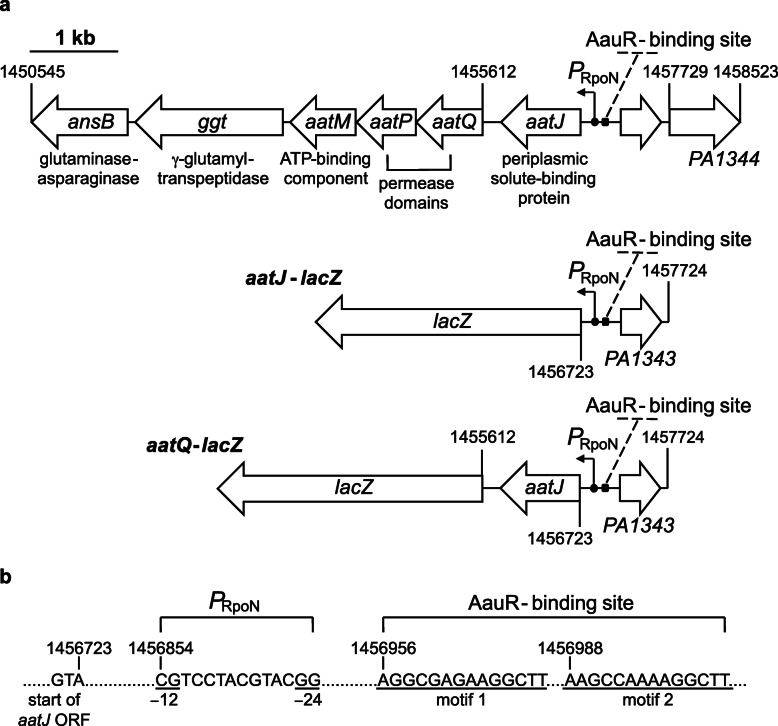
Genetic organization of the *aatJ*-*aatQMP* genes of *P. aeruginosa* PAO1. **a** The uptake of acidic amino acids is believed to mediated through an ABC transporter complex encoded by the *aatJ*-*aatQMP* cluster. Positioned 131–144 bp upstream of the *aatJ* ORF is a putative −12/−24 promoter (*P*_RpoN_), which is the promoter specifically recognized by the sigma factor RpoN. Located upstream of the − 12/− 24 promoter are two sequences or motifs resembling the consensus DNA-binding site for the EBP AauR of *P. putida* KT2440. The presence of these predicted sites is a strong indicator that both AauR and RpoN are involved in the regulation of *aatJ*-*aatQMP* genes, and therefore, the transport of acidic amino acids. To investigate this regulation, two β-galactosidase (LacZ) reporters were constructed and are illustrated: *aatJ*-*lacZ* and *aatQ*-*lacZ*. Although not the subject of the current study, the *ansB* and *ggt* genes potentially form an operon with *aatJ*-*aatQMP*, and collectively, are coordinately regulated in response to acidic amino acids and their amide derivatives in *P. aeruginosa* PAO1. **b** Close-up view of the putative − 12/− 24 promoter (*P*_RpoN_) and AauR-binding site (motifs 1 and 2) located upstream of the *aatJ* ORF in *P. aeruginosa* PAO1

The 5′-regulatory region of the *aatJ*-*aatQMP* genes in *P. aeruginosa* PAO1 possesses putative binding sites for both AauR and RpoN (Fig. 1). One of the putative AauR-binding sites or motifs upstream of *aatJ*-*aatQMP* in *P. aeruginosa* PAO1 is an exact match to the published consensus sequence for AauR of *P. putida* KT2440 [17], and it was previously found that RpoN does bind to the *aatJ* locus in *P. aeruginosa* PA14 through ChIP seq [18]. Collectively, these findings strongly suggest that the *aatJ*-*aatQMP* genes are under the control of AauR and RpoN in *P. aeruginosa* PAO1. However, the *aatJ* and *aatQMP* genes are separated by a 201 bp intergenic region [19]. The presence of such an appreciable intergenic region between the *aatJ* and *aatQMP* genes questions the validity of them forming an operon or being necessarily coordinately regulated. Therefore, in order to have a foundational understanding of AauR regulation in *P. aeruginosa* PAO1, the contributions of both the *aatJ* and *aatQMP* loci, as well as the regulatory *aauR* and *rpoN* genes, towards the utilization of acidic amino acids and their carboxamide counterparts were investigated.

## Results

### The *aauR* and *rpoN* genes are required for optimal growth on L-glutamate and L-glutamine as sole carbon sources

**Fig. 2:**
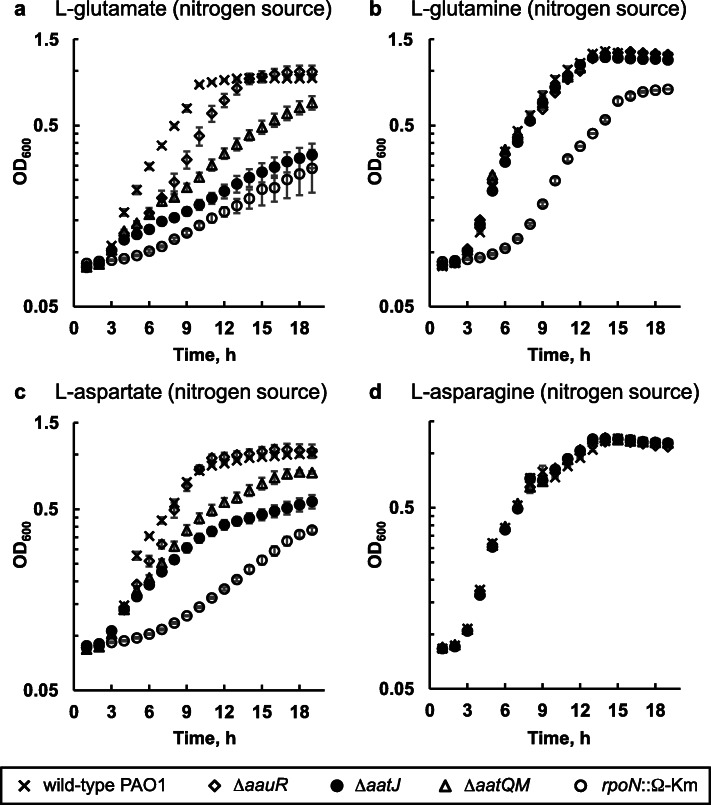
Growth of Δ*aauR, rpoN*::Ω-Km, Δ*aatJ* and Δ*aatQM* mutants of *P. aeruginosa* PAO1 on L-glutamate (**a**), L-glutamine (**b**), L-aspartate (**c**) and L-asparagine (**d**) as nitrogen sources. Bacteria were grown at 37 °C in minimal media supplemented with 20 mM glucose and 10 mM of indicated amino acid as the sole nitrogen source. Data points represent mean values (*n* = 3) ± SD

**Fig. 3:**
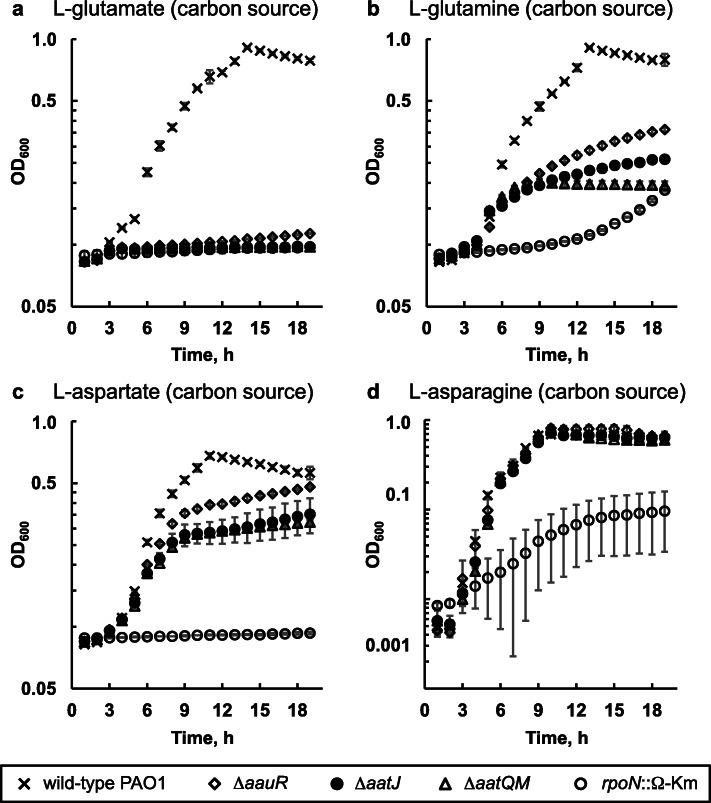
Growth of Δ*aauR, rpoN*::Ω-Km, Δ*aatJ* and Δ*aatQM* mutants of *P. aeruginosa* PAO1 on L-glutamate (**a**), L-glutamine (**b**), L-aspartate (**c**) and L-asparagine (**d**) as carbon sources. Bacteria were grown at 37 °C in minimal media supplemented with 10 mM NH_4_Cl and 20 mM of indicated amino acid as the sole carbon source. Data points represent mean values (*n* = 3) ± SD

**Fig. 4:**
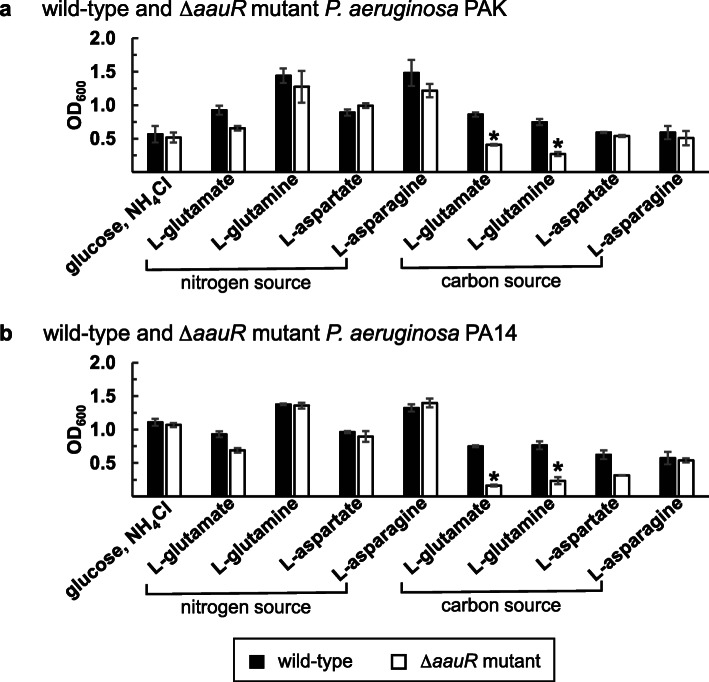
Growth of Δ*aauR* mutants of *P. aeruginosa* strains PAK (**a**) and PA14 (**b**) on acidic amino acids and their amide derivatives as nitrogen or carbon sources. Bacteria were grown at 37 °C for 24 h in minimal media supplemented with 20 mM carbon source and 10 mM nitrogen source as indicated. Data points represent mean values (*n =* 3) ± SD. ANOVA was performed with a Dunnett’s post hoc test (α value, 0.05) to identify significant differences (*P* < 0.0001), which are indicated with asterisks

The *aauR* (*PA1335*), *aatJ* (*PA1342*) and *aatQM* (*PA1341* – *PA1340*) genes were individually deleted from the genome of *P. aeruginosa* PAO1. The resulting markerless Δ*aauR*, Δ*aatJ*, and Δ*aatQM* mutants, in addition to an *rpoN*::Ω-Km mutant and wild-type *P. aeruginosa* PAO1, were then grown in minimal media in which L-glutamate, L-glutamine, L-aspartate or L-asparagine served as the sole source of nitrogen or carbon. As shown in Fig. 2, the growth of the Δ*aauR* mutant did not differ significantly from wild-type *P. aeruginosa* PAO1 when each amino acid served as the sole source of nitrogen. However, growth deficiencies were observed for the Δ*aauR* mutant when either L-glutamate or L-glutamine served as the sole carbon source (Fig. 3). After 18 h of growth on L-glutamate as the sole carbon source, wild-type *P. aeruginosa* PAO1 reached a cell density (OD_600_) of ~ 1.0 while the Δ*aauR* mutant yielded an OD_600_ of ~ 0.1 (Fig. 3a). The Δ*aauR* mutant fared better on L-glutamine as a carbon source, reaching an OD_600_ of ~ 0.3, which was 3-fold lower than that observed for wild-type *P. aeruginosa* PAO1 (Fig. 3b). The final cell densities for wild-type *P. aeruginosa* PAO1 and the Δ*aauR* mutant on L-aspartate as the sole carbon source were similar (OD_600_ of ~ 0.6) (Fig. 3c), and the growth curves for both of these strains were identical to one another when L-asparagine was the carbon source (Fig. 3d). These results suggest that the *aauR* gene of *P. aeruginosa* PAO1 is not essential for the utilization of acidic amino acids and their amide derivatives in general, but instead, is specific towards the utilization of L-glutamate and L-glutamine as sole or preferred carbon sources. Comparable results were observed for Δ*aauR* mutants of *P. aeruginosa* PA14 and PAK, both of which displayed significant growth reductions (> 2-fold) on L-glutamate or L-glutamine as the sole carbon source (Fig. 4).

Growth of the *rpoN*::Ω-Km mutant was significantly decreased compared to that of wild-type *P. aeruginosa* PAO1 under all conditions tested except when L-asparagine served as the sole nitrogen source (Fig. 2d). In the presence of L-glutamate (Fig. 2a) and L-aspartate (Fig. 2c) as nitrogen sources, the *rpoN*::Ω-Km mutant grew to a final OD_600_ of ~ 0.3. The *rpoN*::Ω-Km mutant displayed an extended lag phase on L-glutamine as a nitrogen source (Fig. 2b), but its final OD_600_ was 0.8, which was only slightly below the value of ~ 1.2 observed for wild-*type P. aeruginosa* PAO1 and the Δ*aauR* mutant. When the four amino acids were used as the sole carbon sources, the growth of the *rpoN*::Ω-Km mutant became even more limited. No growth was observed for the *rpoN*::Ω-Km mutant when L-glutamate (Fig. 3a) or L-aspartate (Fig. 3c) was the carbon source. In the presence of L-glutamine (Fig. 3b) or L-asparagine (Fig. 3d) as the carbon source, the cell densities of the *rpoN*::Ω-Km mutant were 5-fold lower than that of wild-type *P. aeruginosa* PAO1. These results support earlier findings demonstrating the essentiality of the *rpoN* gene in the utilization of these amino acids as carbon sources in *P. aeruginosa* PA14 and *P. putida* [20, 21].

### The *aatJ* and *aatQM* genes are essential for optimal growth on L-glutamate, L-glutamine and L-aspartate as sole carbon sources

In the presence of L-glutamate as the sole nitrogen source, the final cell densities of the Δ*aatJ* and Δ*aatQM* mutants were ~ 0.3 and 0.6, respectively, both of which were significantly lower than the OD_600_ of ~ 1.0 observed for the Δ*aauR* mutant and wild-type *P. aeruginosa* PAO1 (Fig. 2a). In contrast, the Δ*aatJ* and Δ*aatQM* mutants displayed wild-type growth when L-glutamine was the only available nitrogen source (Fig. 2b). Hindered growth was observed for the Δ*aatJ* and Δ*aatQM* mutants when L-aspartate was the sole nitrogen source (Fig. 2c), but both mutants grew similar to wild-type *P. aeruginosa* PAO1 in the presence of L-asparagine as the nitrogen source (Fig. 2d). Interestingly, deletions of the *aatJ* and *aatQM* genes had negative effects on the utilization of L-glutamate and L-aspartate as nitrogen sources, which was not observed when the regulatory *aauR* gene was deleted.

There were substantial growth deficiencies observed for the Δ*aatJ* and Δ*aatQM* mutants in the presence of L-glutamate, L-glutamine and L-aspartate as carbon sources (Fig. 3). Both mutants did not grow on L-glutamate as a carbon source (Fig. 3a), and both reached a final OD_600_ of ~ 0.2 on L-glutamine as the sole carbon source (Fig. 3b) compared to ~ 0.9 for wild-type *P. aeruginosa* PAO1. When L-aspartate was the carbon source, the Δ*aatJ* and Δ*aatQM* mutants each yielded an OD_600_ of ~ 0.3 (Fig. 3c), which was about 2-fold lower than that of wild-type *P. aeruginosa* PAO1. The Δ*aatJ* and Δ*aatQM* mutants did, however, display wild-type growth on L-asparagine as the sole carbon source (Fig. 3d).

### Elevated concentrations of L-glutamate are present in the spent medium of the Δ*aauR* mutant compared to wild-type *P. aeruginosa* PAO1

Deletion of the *aauR* gene reduced the growth of *P. aeruginosa* PAO1 on L-glutamate as the sole source of carbon but not nitrogen. This suggested that the uptake or assimilation of L-glutamate was not completely abolished in the Δ*aauR* mutant. To gain further insight into this matter, the depletion of L-glutamate, L-glutamine, L-aspartate and L-asparagine from the growth medium was measured for both the Δ*aauR* mutant and wild-type *P. aeruginosa* PAO1. Cells were grown in glucose-minimal media that was supplemented with one of these four amino acids as the sole nitrogen source at an initial concentration of 20 mM, and the concentration of these four amino acids in the cell-free spent medium were quantified by HPLC at 6.0 and 9.0 h post inoculation.

**Fig. 5:**
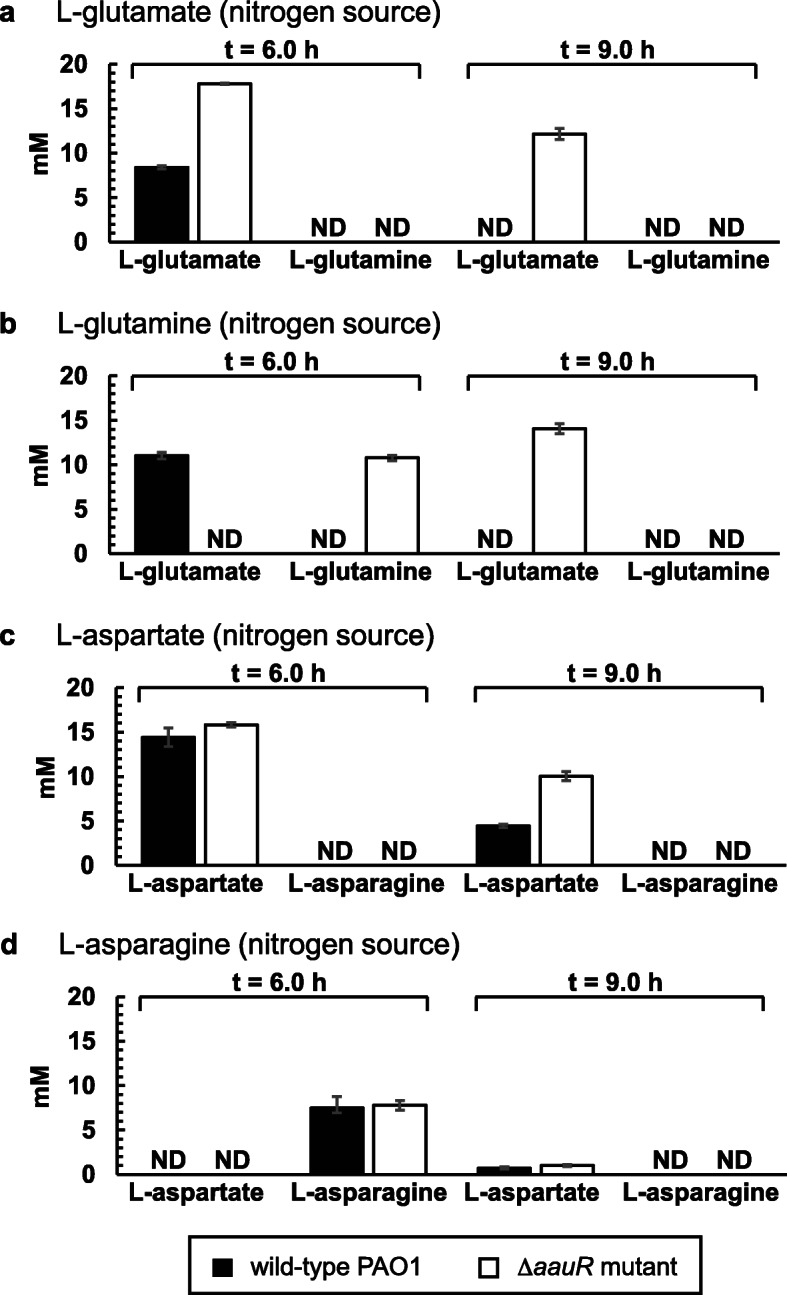
HPLC-measured concentration of L-glutamate (**a**), L-glutamine (**b**), L-aspartate (**c**) and L-asparagine (**d**) in the spent medium of wild-type *P. aeruginosa* PAO1 and the Δ*aauR* mutant at 6.0 and 9.0 h post inoculation. Data points represent mean values ± SD obtained from triplicate bacterial samples grown at 37 °C in glucose-minimal media supplemented with an initial concentration of 20 mM of indicated amino acid. ND denotes not detected

At 6.0 h post inoculation, L-glutamate decreased from an initial concentration of 20 mM to 8.4 ± 0.2 and 17.8 ± 0.1 mM in the spent medium of wild-type *P. aeruginosa* PAO1 and the Δ*aauR* mutant, respectively (Fig. 5a). Three hours later at 9.0 h post inoculation, L-glutamate was not detected in the spent medium from wild-type cells but was still present at a concentration of 12.2 ± 0.6 mM for the Δ*aauR* mutant. The depletion of L-glutamine as the sole nitrogen source was also noticeably different between the two strains (Fig. 5b). At 6.0 h post inoculation, L-glutamine was not detected in the spent medium of wild-type cells but L-glutamate was found to be present at a concentration of 11.0 ± 0.4 mM. Neither L-glutamine nor L-glutamate was observed in the spent medium from wild-type *P. aeruginosa* PAO1 at 9.0 h post inoculation. In sharp contrast, L-glutamine (but not L-glutamate) was detected at a concentration of 10.8 ± 0.3 mM in the spent medium from the Δ*aauR* mutant at 6.0 h post inoculation. Subsequently, at 9.0 h post inoculation, L-glutamine was no longer detected in the spent medium from the Δ*aauR* mutant while L-glutamate was detected at a concentration of 14.1 ± 0.6 mM.

When L-aspartate served as the sole nitrogen source, its concentration in the spent medium was 14.4 ± 1.1 and 15.8 ± 0.2 for wild-type *P. aeruginosa* PAO1 and the Δ*aauR* mutant, respectively, at 6.0 h post inoculation (Fig. 5c). The extracellular concentration of L-aspartate decreased to 4.4 ± 0.2 for wild-type *P. aeruginosa* PAO1 at 9.0 h post inoculation whereas for the Δ*aauR* mutant it was 2-fold higher, yielding a concentration of 10.0 ± 0.5 mM. The depletion of L-asparagine was similar between the Δ*aauR* mutant and wild-type *P. aeruginosa* PAO1 (Fig. 5d). For both strains at 6.0 h post inoculation, extracellular L-asparagine was at a concentration of ~ 8.0 mM and no extracellular L-aspartate was detected. At 9.0 h post inoculation, L-asparagine was completely absent from the spent medium from both strains while L-aspartate was detected at concentrations of ~ 1.0 mM.

### Plasmid-based expression of *aatQMP* or *aatJ*-*aatQMP* in the Δ*aauR* mutant restores its growth to wild-type levels when L-glutamate or L-glutamine served as the sole carbon source

The findings from the aforementioned experiments indicated that the growth deficiencies observed for the Δ*aauR* mutant might be the result of insufficiencies in L-glutamate uptake or transport associated with deregulation of the *aatJ*-*aatQMP* locus. As a first step to address this possibility, the *aatJ*, *aatQMP*, and the *aatJ*-*aatQMP* genes were individually cloned under the *lac* promoter of the expression plasmid pBBR1MCS-5, and the resulting constructs were introduced into the Δ*aauR* mutant. Recombinant strains were subsequently grown in minimal media supplemented with 30 μg mL^− 1^ gentamicin and 20 mM L-glutamate or L-glutamine as the sole carbon source. The Δ*aauR* mutant harboring empty pBBR1MCS-5 and an *aauR*-pBBR1MCS-5 derivative were included in the analysis.

**Fig. 6:**
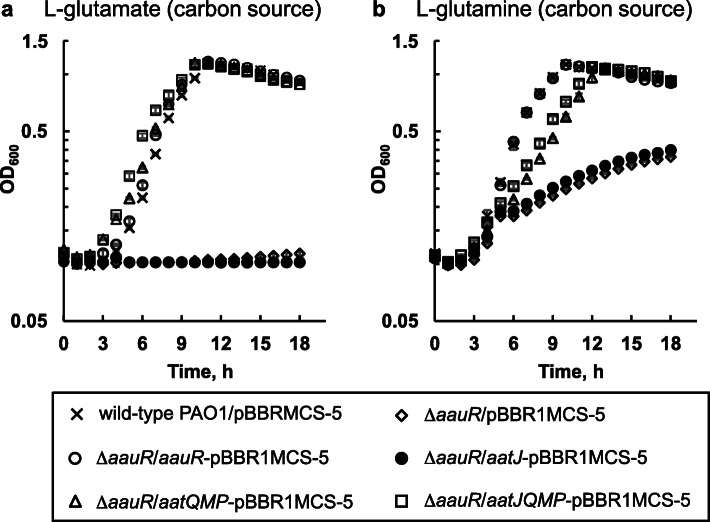
Genetic-complementation experiments for the Δ*aauR* mutant grown on L-glutamate (**a**) and L-glutamine (**b**) as carbon sources. The Δ*aauR* mutant was transformed with expression plasmids encoding for *aauR* (*aauR*-pBBR1MCS-5), *aatJ* (*aatJ*-pBBR1MCS-5), *aatQMP* (*aatQMP*-pBBR1MCS-5) or *aatJQMP* (*aatJQMP*-pBBR1MCS-5). Both wild-type *P. aeruginosa* PAO1 and the Δ*aauR* mutant harboring empty plasmid (pBBR1MCS-5) were included as controls. Bacteria were grown at 37 °C in minimal media supplemented with 30 μg mL^− 1^ gentamicin, 10 mM NH_4_Cl and 20 mM of indicated amino acid as the sole carbon source. Data points represent mean values (*n =* 3) ± SD

The Δ*aauR* mutant transformed with *aauR*-, *aatQMP*-, or *aatJ*-*aatQMP*-pBBR1MCS-5 displayed wild-type levels of growth in the presence of either L-glutamate (Fig. 6a) or L-glutamine (Fig. 6b) as the sole carbon source. In contrast, no growth (OD_600_ < 0.1) was observed for the Δ*aauR* mutant harboring *aatJ*-pBBR1MCS-5 on either amino acid. It is interesting to note that plasmid-based expression of the *aatQMP* genes was sufficient to rescue the L-glutamate and L-glutamine related growth deficiencies of the Δ*aauR* mutant. This suggests that the expression of the *aatQMP* genes, and not *aatJ* nor the entire *aatJ*-*aatQMP* cluster, are the main limiting factor for utilization of L-glutamate and L-glutamine in the absence of the regulatory *aauR* gene.

### The *aauR* and *rpoN* genes are essential for the induction of *aatJ*-*lacZ* and *aatQ*-*lacZ* in response to acidic amino acids and their amide derivatives

Based on the findings of AauR regulation in *P. putida* KT2440 [16], it was expected that expression of the *aatJ*-*aatQMP* genes in *P. aeruginosa* PAO1 would be regulated to some degree by the availability of L-glutamate in a mechanism consisting of the EBP AauR and the sigma factor RpoN. It was uncertain, however, as to whether the expression of the *aatJ*-*aatQMP* genes would be completely dependent on AauR and RpoN, or if these regulatory proteins are only necessary for the upregulation or induction of this locus in response to L-glutamate. To evaluate the expression of the *aatJ*-*aatQMP* genes, two plasmid-based LacZ reporters were constructed: *aatJ*-*lacZ* and *aatQ*-*lacZ*. As shown in Fig. 1, both *aatJ*-*lacZ* and *aatQ*-*lacZ* have the same ~ 1000 bp 5′-regulatory region immediately upstream of the *aatJ* ORF. The *aatQ*-*lacZ* also possesses the *aatJ* ORF and the 201 bp intergenic region between the *aatJ* and *aatQ* ORFs.

The *aatJ*-*lacZ* and *aatQ*-*lacZ* reporters were introduced into the Δ*aauR* and *rpoN*::Ω-Km mutants, as well as wild-type *P. aeruginosa* PAO1. Recombinant strains were grown in L-alanine-minimal media to an OD_600_ of 0.2, and subsequently challenged with the addition of 20 mM of succinate, α-KG, L-arginine, L-histidine, L-glutamate, L-aspartate, L-glutamine or L-asparagine. Because L-alanine was reported to have minimal influence in catabolite repression and global-gene regulation in *Pseudomonas* [22, 23], it was an ideal choice for establishing a baseline level of expression for the *aatJ*-*lacZ* and *aatQ*-*lacZ* reporters while simultaneously serving as a permissible or growth-sustaining nutrient for the Δ*aauR* and *rpoN*::Ω-Km mutants. Representative C_4_- and C_5_-dicarboxylates were included as substrates in the analysis due to the fact that AauR is a predicted member of the DctD-family of EBPs. In addition, L-glutamate is an intermediate in the catabolism of L-arginine and L-histidine [24, 25], so both of these amino acids were included as substrates in these assays. Because a lag phase of ~ 3.0 h was observed for wild-type *P. aeruginosa* PAO1 when grown on acidic amino acids and their amide derivatives, LacZ activity was measured near the beginning and end of this phase, i.e., 1.0 and 3.0 h post addition of substrate. LacZ activity is reported in Miller Units (MU).

**Fig. 7:**
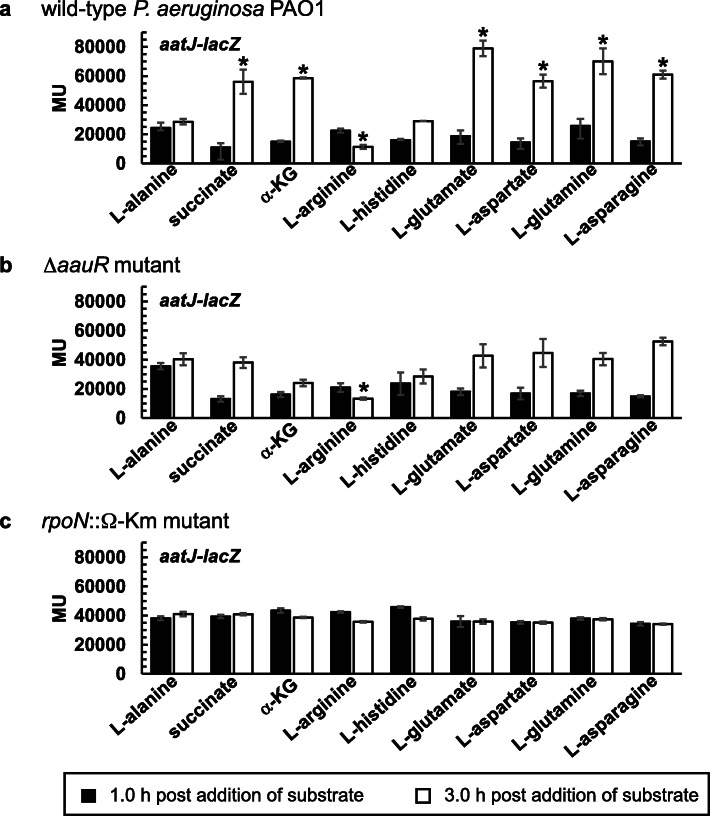
LacZ activity of *aatJ*-*lacZ* in wild-type *P. aeruginosa* PAO1 (**a**), the Δ*aauR* mutant (**b**) and the *rpoN*::Ω-Km mutant (**c**). Bacteria were grown in alanine-minimal media to an OD_600_ of ~ 0.2 and then challenged with 20 mM of indicated substrate. LacZ activity was subsequently measured at 1.0 and 3.0 h post addition of substrate and is reported in Miller Units (MU). Data points represent mean values (*n =* 3) ± SD. For each strain or mutant, an ANOVA was performed with a Dunnett’s post hoc test (α value, 0.05) to identify significant changes in the expression of *aatJ*-*lacZ* relative to the alanine-treated control. Significant differences (*P* < 0.0001) are indicated with asterisks

The expression levels for *aatJ*-*lacZ* in response to L-alanine as the sole carbon source were ~ 25,000 MU in wild-type *P. aeruginosa* PAO1 at both the 1.0 and 3.0 h timepoints (Fig. 7a). Expression of *aatJ*-*lacZ* in response to L-alanine was only slightly higher for the Δ*aauR* (Fig. 7b) and *rpoN*::Ω-Km (Fig. 7c) mutants, yielding LacZ activities of ~ 35,000 MU for both timepoints. The expression levels for *aatJ*-*lacZ* in the sole presence of L-alanine were not statistically different among the three strains, and thus, served as the baseline for all subsequent comparisons.. When challenged with L-glutamate, L-aspartate, L-glutamine or L-asparagine, the expression levels of *aatJ*-*lacZ* increased by > 2-fold in wild-type *P. aeruginosa* PAO1 at 3.0 h post addition (Fig. 7a) whereas no such induction was observed for the Δ*aauR* (Fig. 7b) or *rpoN*::Ω-Km mutant (Fig. 7c). The expression levels of *aatJ*-*lacZ* did not significantly increase at 1.0 h post addition for any tested substrate or strain.

Expression of *aatJ*-*lacZ* was significantly influenced by the presence of succinate, α-KG and L-arginine in wild-type *P. aeruginosa* PAO1 (Fig. 7a). At 3.0 h post addition of succinate or α-KG, expression of *aatJ*-*lacZ* increased by 2-fold (~ 55,000 MU) in wild-type *P. aeruginosa* PAO1. The presence of L-arginine actually repressed the expression of *aatJ*-*lacZ*, yielding LacZ activities of ~ 15,000 MU for wild-type *P. aeruginosa* PAO1 at the 3.0 timepoint. Similar repression of *aatJ*-*lacZ* by L-arginine was observed for the Δ*aauR* mutant (Fig. 7b). The changes or fluctuations in the expression of *aatJ*-*lacZ* caused by succinate, α-KG or L-arginine were not observed in the *rpoN*::Ω-Km mutant (Fig. 7c). Lastly, the presence of L-histidine did not significantly affect the expression of *aatJ*-*lacZ* in any of the three investigated strains.

**Fig. 8:**
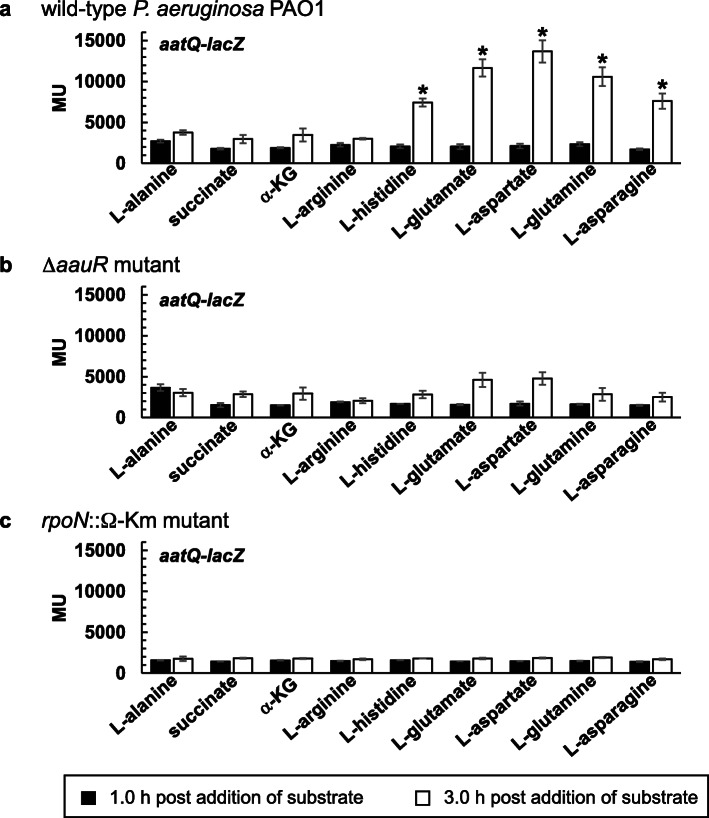
LacZ activity of *aatQ*-*lacZ* in wild-type *P. aeruginosa* PAO1 (**a**), the Δ*aauR* mutant (**b**) and the *rpoN*::Ω-Km mutant (**c**). Bacteria were grown in alanine-minimal media to an OD_600_ of ~ 0.2 and then challenged with 20 mM of indicated substrate. LacZ activity was subsequently measured at 1.0 and 3.0 h post addition of substrate and is reported in Miller Units (MU). Data points represent mean values (*n =* 3) ± SD. For each strain or mutant, an ANOVA was performed with a Dunnett’s post hoc test (α value, 0.05) to identify significant changes in the expression of *aatQ*-*lacZ* relative to the alanine-treated control. Significant differences (*P* < 0.0001) are indicated with asterisks

The expression levels for *aatQ*-*lacZ* were significantly lower than that of *aatJ*-*lacZ* for all tested substrates in all three strains (Fig. 8). In wild-type *P. aeruginosa* PAO1 (Fig. 8a), the expression levels for *aatQ*-*lacZ* in response to L-alanine as the sole carbon source generated LacZ activities of ~ 3500 MU for both the 1.0 and 3.0 h timepoints. This value increased by > 2-fold at 3.0 h post addition of L-glutamate, L-aspartate, L-glutamine, L-asparagine, or unexpectedly, L-histidine. Unlike that of *aatJ*-*lacZ*, the addition of succinate, α-KG or L-arginine had no significant effect on the expression of *aatQ*-*lacZ* in wild-type *P. aeruginosa* PAO1. Expression of *aatQ*-*lacZ* in the Δ*aauR* (Fig. 8b) and *rpoN*::Ω-Km (Fig. 8c) mutants was not significantly affected by the addition of any substrate. The average LacZ activities for *aatQ*-*lacZ* in these mutants ranged from 2000 to 4500 MU.

## Discussion

### The transporter AatJQMP has a fundamental role in the utilization of L-glutamate and L-glutamine in *P. aeruginosa* PAO1

The genome of *P. aeruginosa* PAO1 encodes for three putative glutamate transporters: the ABC-transporter complex AatJQMP (PA1339 – PA13342), the sodium-glutamate symporter GltS (PA3176), and the proton-glutamate symporter GltP (PA5479). Deletion of the *aatJ* or *aatQM* genes in *P. aeruginosa* PAO1 significantly reduced the growth of this bacterium on L-glutamate as either the sole source of nitrogen or carbon (Figs. 2a and 3a); although as a sole carbon source, growth was more severely hindered. Similar growth deficiencies on L-glutamate have been observed for *aatQMP*-transposon mutants of *P. aeruginosa* PAK [26]. It would appear that the importance of AatJQMP in L-glutamate utilization is not restricted to PAO1, but is likely common among other various strains and isolates of *P. aeruginosa*. The roles of GltS, GltP and other dicarboxylate-transport proteins in L-glutamate utilization have yet to be resolved, but the presence of these transporters would explain the lingering or residual growth observed for the Δ*aatJ* and Δ*aatQM* mutants of *P. aeruginosa* PAO1 on L-glutamate. Lastly, it should be mentioned that transposon insertions of the *aatJ*, *aatQ*, and *aatP* genes were previously reported to abolish the growth of *P. aeruginosa* PAO1 on D-glutamate as the sole carbon and nitrogen source [27]. This suggest that the transporter AatJQMP is not stereospecific, and thus, plays a central role in the utilization of both L- and D-isomers of glutamate in *P. aeruginosa* PAO1.

The *aatJ-aatQMP* genes were required for optimal growth of *P. aeruginosa* PAO1 on L-glutamine as the sole source of carbon but not nitrogen (Figs. 2b and 3b). The *PA5073* – *PA5076* operon of *P. aeruginosa* PAO1 encodes for a putative glutamine ABC-transporter complex, but a previous analysis of transposon mutants indicates that this locus is not required for growth on any of the twenty common amino acids [26]. Nonetheless, it is still plausible that L-glutamine enters the cell through PA5073 – PA5076 and then undergoes transamination and/or deamidation to yield L-glutamate. Another route for assimilation of L-glutamine involves deamidation in the periplasm catalyzed by the glutaminase-asparaginase AnsB (PA1337) [28] with the subsequent transport of the L-glutamate product via AatJQMP, GltS, GltP and/or additional dicarboxylate-transport proteins. Data from the HPLC analysis (Fig. 5b) supports the existence of this extracellular metabolic conversion. Notably, because the periplasmic deamidation of L-glutamine liberates ammonia, a readily consumable nitrogen source, the bacterium does not have a strict requirement for the uptake of L-glutamine directly or the L-glutamate product to fulfill its nitrogen demands. This would account for the unperturbed growth observed for the Δ*aatJ* and Δ*aatQM* mutants of *P. aeruginosa* PAO1 on L-glutamine as a source of nitrogen but not carbon (Figs. 2b and 3b); the latter of which requires the L-glutamate product as a precursor for cellular biosynthesis. A similar result was observed for *P. aeruginosa* PAK in which a transposon mutation of the *aatM* gene eliminated its growth on L-glutamine as a source of carbon but not nitrogen [26].

### The transporter AatJQMP affects the utilization of L-aspartate but not L-asparagine in *P. aeruginosa* PAO1

Growth was observed for both the Δ*aatJ* and Δ*aatQM* mutants on L-aspartate as a nitrogen or carbon source, but the final cell densities for these mutants were 2-fold lower than that of wild-type *P. aeruginosa* PAO1 (Figs. 2c and 3c). This indicates that the transporter AatJQMP is required for optimal utilization of L-aspartate as a preferred or sole nutrient. In an earlier study, data from LacZ assays and microarray analysis clearly demonstrated that exogenous L-aspartate induced expression of the *dctA* and *dctPQM* genes in *P. aeruginosa* PAO1 [29]. These previous results suggest that the C_4_-dicarboxylate transporters DctA and DctPQM might facilitate the uptake of L-aspartate, and therefore, would give reason as to why the Δ*aatJ* and Δ*aatQM* mutations had a less deleterious effect in the utilization of L-aspartate compared to that of L-glutamate, a C_5_-dicarboxylate. For comparison purposes, neither L-aspartate nor L-glutamate induced expression of the C_5_-dicarboxylate transporter gene *PA5530* [13, 29].

The utilization of L-asparagine is not dependent on AatJQMP in *P. aeruginosa* PAO1. Analogous to that of L-glutamine, one might expect that exogenous L-asparagine is deaminated in the periplasm through the actions of AnsB, and the resulting L-aspartate transported intracellularly via AatJQMP, DctA and DctPQM. However, results from our HPLC analysis indicated that very little L-aspartate (< 1.0 mM) accumulated in the extracellular milieu of *P. aeruginosa* PAO1 when fed an initial concentration of 20 mM L-asparagine as the sole nitrogen source (Fig. 5d). This is in sharp contrast to the > 10 mM L-glutamate that amassed in the spent medium when cells were given a starting concentration of 20 mM L-glutamine as the sole nitrogen source (Fig. 5b). In a previous study, exogenous L-asparagine was shown to induce expression of two transporter-gene *lacZ* fusions, *PA5530*-*lacZ* and *PA2252*-*lacZ* [29]. The *PA2252* gene encodes for a putative sodium-amino acid symporter and potentially forms an operon with the *ansA* gene, encoding for a cytoplasmic glutaminase-asparaginase. The direct transport of L-asparagine through PA5530 and/or PA2252 followed by AnsA-catalyzed deamidation would account for the non-essential nature of the *aatJ-aatQMP* genes in the utilization this amino acid in *P. aeruginosa* PAO1.

### AauR and RpoN are essential for the induction of *aatJ*-*aatQMP* in response to acidic amino acids and their amide derivatives *P. aeruginosa* PAO1

The results from the LacZ assays for *aatJ*-*lacZ* and *aatQ*-*lacZ* revealed several key factors surrounding the expression and regulation of the *aatJ*-*aatQMP* genes in *P. aeruginosa* PAO1. First, expression of both *aatJ*-*lacZ* and *aatQ*-*lacZ* were induced or upregulated in the presence of exogenous L-glutamate (Figs. 7a and 8a), suggesting that the expression of the *aatJ*-*aatQMP* genes are coordinately upregulated in response to this amino acid. Such coordinated upregulation is not limited to L-glutamate, because the presence of L-aspartate, L-glutamine and L-asparagine also induced expression of *aatJ*-*lacZ* and *aatQ*-*lacZ* (Figs. 7a and 8a). Second, the upregulation of *aatJ*-*lacZ* and *aatQ*-*lacZ* in response to these four amino acids was completely dependent on the *aauR* and *rpoN* genes. This is consistent with the EBP AauR and the sigma factor RpoN being directly involved in the activation of the *aatJ*-*aatQMP* genes in *P. aeruginosa*.

Third, both *aatJ*-*lacZ* and *aatQ*-*lacZ* were still expressed in the absence of (*i*) the *aauR* and *rpoN* genes, and (*ii*) exogenous L-glutamate, L-aspartate, L-glutamine or L-asparagine. These expression levels, however, were less than that of their induced states. The same appears to be true for *P. putida* KT2440 in which deletion of the *aauR* gene did not eliminate expression of an *aatJ*-*lacZ* reporter, but instead, reduced its expression by ~ 50% in response to L-glutamate [16]. Fourth and last, the expression levels of *aatJ*-*lacZ* were significantly greater (> 5-fold) than that of *aatQ*-*lacZ* under both inducing and non-inducing conditions. Collectively, these findings indicate that although the *aatJ*-*aatQMP* genes in *P. aeruginosa* PAO1 are coordinately upregulated by AauR-RpoN in response to certain amino acids, they are also expressed independently of these two regulatory proteins, including a potential mechanism in which the *aatJ* gene is expressed separately or distinctly from that of *aatQMP*.

The multivariate expression surrounding the *aatJ*-*aatQMP* genes of *P. aeruginosa* PAO1 is evident in the response of *aatJ*-*lacZ* and *aatQ*-*lacZ* to various substrates (Figs. 7 and 8). The presence of L-arginine repressed expression of *aatJ*-*lacZ* but not *aatQ*-*lacZ* whereas exogenous L-histidine induced expression of the latter and not the former. An earlier study did report that the transcript levels of *aatJ* in *P. aeruginosa* PAO1 were ~ 2-fold higher for cells grown in L-glutamate compared to L-arginine as the sole nitrogen source [27]. However, the analysis of the ArgR regulon of *P. aeruginosa* PAO1 revealed that the transcript levels for *aatJ* were not significantly different between cells grown in L-glutamate versus a mixture of L-glutamate and L-arginine [24]. Therefore, it would appear that L-arginine represses the expression of *aatJ* when present as the sole nutrient, i.e., in the absence of L-glutamate. ArgR was reported to repress genes associated with glutamate metabolism [24], so it possible that *aatJ* is also a target of negative control by this regulator. In the presence of both L-arginine and L-glutamate, expression of *aatJ* might be a competition between repression and activation via ArgR and AauR, respectively. Curiously, the presence of dicarboxylates such as succinate and α-KG, which are preferred carbon sources in *Pseudomonas* [3, 4], caused ~ 2-fold increase in the expression levels of *aatJ*-*lacZ* and *aatQ*-*lacZ*. An earlier transcriptomic study did identify the *aatJ* gene as a potential regulatory target of catabolite repression in *P. aeruginosa* PAO1 [30], and results from ChIP seq showed that the sigma factor FecI binds to the *aatJ* locus in *P. aeruginosa* PA14 [18] . Further investigation is needed to determine how such control points and regulatory factors contribute to the expression of the *aatJ*-*aatQMP* genes in *P. aeruginosa*.

### The EBP AauR plays a pivotal role in the utilization of L-glutamate and L-glutamine as sole or preferred nutrients

Two significant growth deficiencies were observed for the Δ*aauR* mutant of *P. aeruginosa* PAO1. The Δ*aauR* mutant did not grow on L-glutamate as the sole carbon source (Fig. 3a), and it displayed a ~ 2-fold growth reduction on L-glutamine as the sole carbon source (Fig. 3b). The results of the genetic-complementation experiments (Fig. 6) and LacZ assays (Figs. 7b and 8b) suggest that these growth deficiencies were due to inadequate expression of the *aatQMP* genes. Namely, plasmid-based expression of the *aatQMP* genes restored the growth of the Δ*aauR* mutant to wild-type levels, and expression of *aatQ*-*lacZ* was downregulated by > 2-fold in the absence of the *aauR* gene. In comparison, plasmid-based expression of *aatJ* did not significantly impact or improve the growth of the Δ*aauR* mutant, and even though expression of *aatJ*-*lacZ* was not induced in the Δ*aauR* mutant, the non-induced or basal expression levels were still substantial and greater than that of *aatQ*-*lacZ*. It is AauR-mediated upregulation of the *aatQMP* genes, and not *aatJ*, which is a decisive factor in the utilization of L-glutamate and L-glutamine as sole or preferred nutrients in *P. aeruginosa* PAO1. Unlike the Δ*aatJ* and Δ*aatQM* mutants, the growth of the Δ*aauR* mutant did not differ significantly from wild-type *P. aeruginosa* PAO1 when L-glutamate served as the only nitrogen source (Fig. 2a) or L-aspartate served as the sole nitrogen or carbon source (Figs. 2c and 3c). The upregulation of the *aatJ*-*aatQMP* genes via AauR-RpoN is non-essential under such conditions.

### The sigma factor RpoN is necessary for the utilization of acidic amino acids and their amide derivatives

The *rpoN*::Ω-Km mutant of *P. aeruginosa* PAO1 exhibited growth deficiencies on both L-glutamate and L-glutamine when either amino acid served as the sole source of carbon or nitrogen (Figs. 2 and 3). Based on the results of the LacZ assays (Figs. 7c and 8c), the observed growth deficiencies of the *rpoN*::Ω-Km mutant were likely caused to some degree by the downregulation of the *aatJ*-*aatQMP* genes. In addition, the *rpoN*::Ω-Km mutant displayed growth deficiencies on L-aspartate as either a nitrogen or carbon source (Figs. 2c and 3c). As noted earlier, the dicarboxylate-transport proteins DctA and DctPQM have been implicated in the transport of L-aspartate [29], and the expression of the corresponding *dctA* and *dctPQM* genes are dependent on RpoN [11]. The downregulation or reduced expression of the *aatJ*-*aatQMP*, *dctA* and *dctPQM* genes is expected to have a negative effect in the utilization of L-aspartate as observed for the *rpoN*::Ω-Km mutant. The possible role or function of RpoN in the utilization of L-asparagine is less clear. While RpoN was required for growth on L-asparagine as the sole carbon source (Fig. 3d), it was unnecessary when this amino acid was the only available nitrogen source (Fig. 2d). One potential contributing factor is the transporter PA5530, whose expression is positively regulated by both RpoN and L-asparagine [13, 29]. In contrast, the *PA2252*-*ansA* genes, which have also been associated with L-asparagine utilization [29], are neither predicted nor experimentally-proven targets of RpoN regulation in *P. aeruginosa*.

The growth deficiencies observed for the *rpoN*::Ω-Km mutant can be attributed to some extent to the downregulation of specific target genes, but it is plausible that pleiotropic effects were also an underlying factor. The RpoN regulon of *P. aeruginosa* is extensive - second only to that of the housekeeping sigma factor RpoD [18]. The transcription of > 500 genes are affected by RpoN [18], which regulates a consortium of diverse cellular processes such as motility [31], quorum sensing [32, 33], transport of dicarboxylates [11, 13], catabolite repression and nitrogen assimilation [34, 35]. Consequently, the deregulation of core metabolic and assimilatory pathways is expected to hinder or limit the overall growth of the *rpoN*::Ω-Km mutant. The expression patterns of *aatJ*-*lacZ* (Fig. 7c) and *aatQ*-*lacZ* (Fig. 8c) in the *rpoN*::Ω-Km mutant is also suggestive of such deregulation. For example, exogenous L-arginine repressed the expression of *aatJ*-*lacZ* in the Δ*aauR* mutant and wild-type *P. aeruginosa* PAO1, whereas in the *rpoN*::Ω-Km mutant, it had no effect. Furthermore, both *aatJ*-*lacZ* and *aatQ*-*lacZ* were unresponsive in the *rpoN*::Ω-Km mutant.

### The scope and magnitude of AauR regulation in *P. aeruginosa* PAO1

The last 38 amino acid residues of the C-terminus of AauR form a putative FIS-type helix-turn-helix (HTH) motif. This FIS-type HTH or DNA-binding domain is 79% identical between the AauR proteins of *P. aeruginosa* PAO1 and *P. putida* KT2440, suggesting that these two EBPs recognize similar if not identical DNA-binding sites. Located 131–144 and 233–278 bp upstream of the *aatJ* ORF in *P. aeruginosa* PAO1 are sequences resembling the unique − 12/− 24 promoter recognized by RpoN and the consensus DNA-binding site for AauR, respectively (Fig. 1). Apart from *aatJ*, no other genes in *P. aeruginosa* PAO1 possess sequences matching the consensus DNA-recognition site of AauR in their 5′-regulatory regions. Biochemical characterization of AauR from *P. aeruginosa* PAO1 should reveal the exact DNA-binding site for this regulator and will be invaluable in generating a more definitive answer as to the number and range of genes that are directly governed by this EBP.

**Fig. 9:**
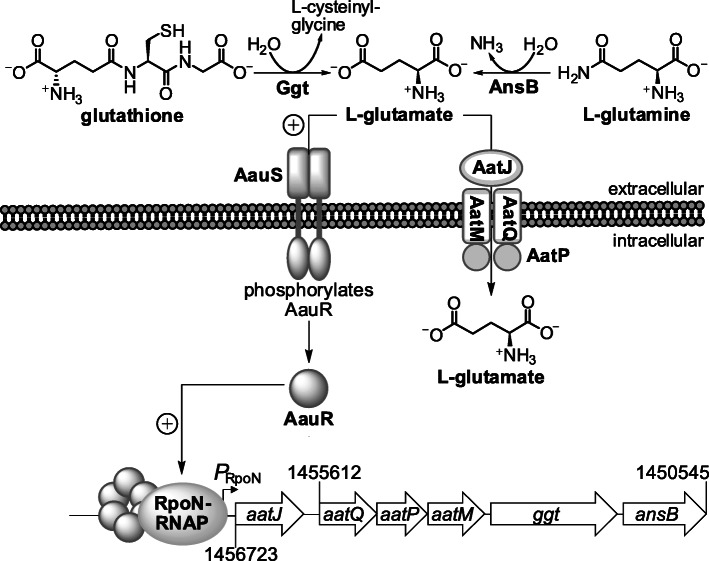
The AauSR TCS in the utilization of L-glutamate and L-glutamine in *P. aeruginosa* PAO1. Extracellular L-glutamate is sensed by the histidine kinase AauS, which subsequently phosphorylates its cognate partner EBP AauR. Phosphorylated AauR (shown as a hexamer [6]) interacts with the RpoN-RNA polymerase complex and mediates the transcriptional activation of the *aatJ*-*aatQMP* genes from the − 12/− 24 promoter (*P*_RpoN_). The *ansB* and *ggt* genes are potentially transcribed with *aatJ*-*aatQMP*, but experimental verification is needed. The periplasmic glutaminase-asparaginase AnsB catalyzes the deamidation of L-glutamine to yield L-glutamate whereas the metabolic role of Ggt is unknown but might involve the liberation of L-glutamate from the hydrolysis of γ-glutamyl compounds such as glutathione

While the *aatJ*-*aatQMP* genes were the focus of the current study, it is probable that AauR and RpoN regulate the expression of two other genes associated with this locus (Figs. 1 and 9). Namely, the *aatJ*-*aatQMP* genes might form an operon with genes encoding for the periplasmic glutaminase-asparaginase AnsB and a periplasmic gamma-glutamyltranspeptidase (Ggt) [19]. As mentioned earlier, the utilization of L-glutamine is thought to involve AnsB, which catalyzes the deamidation of this amino acid in the periplasm to liberate L-glutamate [28]. Deletion of the *aauR* did hinder the growth of *P. aeruginosa* PAO1 on L-glutamine as the sole carbon source, but the total disappearance or depletion of exogenously fed L-glutamine was only delayed and not abolished in the Δ*aauR* mutant, arguing that the deamidation of L-glutamine is not strictly dependent on AauR in *P. aeruginosa* PAO1. Interestingly, growth on L-asparagine but not L-glutamine was significantly reduced in an Δ*ansA* Δ*ansB* double mutant of *P. aeruginosa* PAO1 [29]. This suggests that additional glutaminases apart from the cytoplasmic AnsA and periplasmic AnsB are involved in the deamidation and metabolism of L-glutamine in *P. aeruginosa*. The enzyme Ggt catalyzes the transfer of γ-glutamyl groups from donor molecules, most notably glutathione, to target or acceptor substrates consisting of amino acids, peptides or even water. Although Ggt has been implicated in the metabolism of glutathione and cysteine in some bacteria [36], little is known on the biological importance of this enzyme in *Pseudomonas*, including any potential role it may have in glutamate-related metabolism.

### The sensor histidine kinase AauS is hypothesized to be essential for optimal utilization of L-glutamate and L-glutamine in *P. aeruginosa*

The sensor histidine kinase AauS and the EBP AauR form a putative DctBD-type TCS. The transcriptional activity of AauR is, therefore, believed to be controlled through phosphorylation catalyzed by AauS. Because AauS controls the transcriptional activity of AauR, it is reasonable to predict that optimal utilization of L-glutamate and L-glutamine in *P. aeruginosa* will be dependent on AauS. Indeed, an earlier study reported that the growth phenotypes of *aauS* and *aauR* mutants of *P. putida* KT2440 were similar to one another [16]. Thus, comparable results are expected in *P. aeruginosa*.

The results of the LacZ assays strongly suggest that the AauSR TCS regulates the expression of the *aatJ*-*aatQMP* genes in response to L-glutamate, L-aspartate, L-glutamine and L-asparagine. The question then becomes: what is the substrate specificity of AauS? Does AauS recognize each amino acid, or are only the acidic amino acids capable of binding to and stimulating the histidine kinase activity of AauS? In the latter scenario, the periplasmic deamidation of L-glutamine and L-asparagine would generate the requisite acidic amino acid substrates for AauS. Another potential and unexpected substrate of AauS is that of L-histidine, which was found to induce expression of *aatQ-lacZ* in a manner dependent on AauR. Intracellular L-glutamate is an intermediate in the catabolism of L-histidine, and studies involving *P. putida* and *Rhizobium leguminosarum* have indicated that L-glutamate efflux may occur under certain conditions [17, 37, 38]. Perhaps the AauSR TCS of *P. aeruginosa* facilitates the recapture of effluxed or escaped L-glutamate during L-histidine catabolism. Lastly, given that the transporter AatJQMP is required for growth on both D- and L-glutamate in *P. aeruginosa*, it would suggest that AauS is also not stereospecific, i.e., either isomer of glutamate is sufficient for stimulating AauS activity. Current efforts are underway to determine the substrate specificity and biological significance of AauS in *P. aeruginosa* PAO1.

## Conclusions

The results of this study provide some much-needed insight into the biological function and significance of the EBP AauR in *P. aeruginosa* (Fig. 9). In response to extracellular acidic amino acids and their amide derivatives, AauR and the sigma factor RpoN upregulate the expression of the *aatJ*-*aatQMP* genes, encoding for an ABC-transporter complex that mediates the uptake of L-glutamate and L-aspartate. Specifically, it is the AauR-RpoN mediated upregulation of the *aatQMP* genes (and not *aatJ*) that is a crucial, growth-limiting step in the utilization of L-glutamate and L-glutamine as sole or preferred nutrients in *P. aeruginosa* PAO1. The upregulation of *aatJ*-*aatQMP* via AauR-RpoN was not essential for the utilization of L-aspartate or L-asparagine, suggesting that this particular control mechanism is more influential or important towards the catabolism of the L-glutamate/L-glutamine pair.

## Methods

### Bacteria and bacteriological media

Bacteria used in the study are given in Table 1. Bacteria were grown in Lennox broth (LB) or minimal media (22 mM KH_2_PO_4_, 42 mM Na_2_HPO_4_, 8.6 mM NaCl, 2.0 mM MgSO_4_, 5.0 μM FeSO_4_, pH 7.0), which was supplemented with carbon and nitrogen sources as indicated per experiment described below. Solid bacteriological media was prepared with the addition of BD Difco™ agar at 15 g L^− 1^. Plasmids and chromosomal-genetic markers were selected with the following antibiotics at the indicated final concentrations in μg mL^− 1^: carbenicillin (200 for *P. aeruginosa*, 100 for *E. coli*); gentamicin (30 for *P. aeruginosa*, 25 for *E coli*; kanamycin (50 for *E. coli*).

**Table 1:** Bacteria used in the current study

Strains	Relevant characteristics	Source
*Pseudomonas aeruginosa*		
PAO1	wild type	[32]
∆*aauR* PAO1	∆*aauR* derivative of PAO1	This study
∆*aatQM* PAO1	∆*aatQM* derivative of PAO1	This study
∆*aatJ* PAO1	∆*aatJ* derivative of PAO1	This study
*rpoN*:Ω-Km PAO1	Δ*rpoN* derivative of PAO1	[32]
PA14	wild type	[39]
∆*aauR* PA14	∆*aauR* derivative of PA14	This study
PAK	wild type	[40]
∆*aauR* PAK	∆*aauR* derivative of PAK	This study
*Escherichia coli* MG1655	F^−^ λ^−^ *ilvG*^−^ *rfb-50 rph-1*	[41]
*Escherichia coli* Top10^a^	F^*−*^ *mcrA Δ(mrr-hsdRMS-mcrBC) φ80lacZΔM15 ΔlacX74 nupG recA1 araD139 Δ (ara-leu)7697 galE15 galK16 rpsL(Str*^*r*^*) endA1 λ*^*−*^	Invitrogen

^a^Used for cloning purposes and plasmid maintenance

### Molecular biology methods

Plasmids and oligonucleotides used in the study are given in Tables 2 and 3, respectively. Molecular biology enzymes (restriction endonucleases, DNA polymerases and ligases) were purchased from New England BioLabs (Ipswich, Massachusetts, USA) whereas the purification of nucleic acids was completed using commercially available kits from Promega (Madison, Wisconsin, USA). Cloned DNA was sequenced to confirm its identity.

**Table 2:** Plasmids used in the current study

Plasmids	Relevant characteristics^**a**^	Source
pCR-Blunt	Cloning plasmid; Km^r^	Invitrogen
pBBR1MCS-5	Broad-host-range plasmid; Gm^r^	[42]
pDONR221	Gateway cloning plasmid; Km^r^	Invitrogen
pEX18ApGW	Gene-deletion plasmid for *P. aeruginosa*; Cb^r^	[43]
∆P*lac*-pBBR1MCS-5	pBBR1MCS-5 minus *lac* promoter; Gm^r^	[13]
pJMS02	*aauR* in pCR-Blunt; Km^r^	This study
pJMS03	*aauR* in pBBR1MCS-5; Gm^r^	This study
pJMS04	*aatQMP* in pCR-Blunt; Km^r^	This study
pJMS05	*aatQMP* in pBBR1MCS-5; Gm^r^	This study
pJMS07	*aatJ-lacZ* in pCR-Blunt; Km^r^	This study
pJMS08	*aatQM-lacZ* in pCR-Blunt; Km^r^	This study
pJMS09	*aatJ-lacZ* in ∆P*lac*-pBBR1MCS-5; Gm^r^	This study
pJMS10	*aatQM-lacZ* in ∆P*lac*-pBBR1MCS-5; Gm^r^	This study
pJMS18	*aatJQMP* in pCR-Blunt; Km^r^	This study
pJMS19	*aatJQMP* in pBBR1MCS-5; Gm^r^	This study
pJMS20	*aatJ* in pCR-Blunt; Km^r^	This study
pJMS21	*aatJ* in pBBR1MCS-5; Gm^r^	This study
pJMS298	*aauR::FRT-aacC1-FRT* in pDONR221; Km^r^ Gm^r^	This study
pJMS299	*aauR::FRT-aacC1-FRT* in pEX18ApGW; Cb^r^ Gm^r^	This study
pJMS300	*aatJ::FRT-aacC1-FRT* in pDONR221; Km^r^ Gm^r^	This study
pJMS301	*aatJ::FRT-aacC1-FRT* in pEX18ApGW; Cb^r^ Gm^r^	This study
pJMS302	*aatQM::FRT-aacC1-FRT* in pDONR221; Km^r^ Gm^r^	This study
pJMS303	*aatQM::FRT-aacC1-FRT* in pEX18ApGW; Cb^r^ Gm^r^	This study

^a^Note that Cb^r^, Gm^r^ and Km^r^ denote resistance to carbenicillin, gentamicin and kanamycin, respectively

**Table 3:** Oligonucleotides used in the current study

Oligonucleotides	Sequence	Purpose^**a**^
BL342.r	gcagttatttttgacaccagaccaactggta	*E. coli lacZ*
JS05.r	tgaatccgtaatcatggtcatgttggggtttcccctcgg	*aatQ*-*lacZ*
JS06.f	ccgaggggaaaccccaacatgaccatgattacggattcactgg	*E. coli lacZ* (*aatQ* overlap)
JS42.f	ccgaggggaaaccccaacatgaccatgattacggattcactgg	*E. coli lacZ* (*aatJ* overlap)
JS43.r	tgaatccgtaatcatggtcatgaatttcttcctcgacttgttttg	*aatJ*-*lacZ*
JS35P.f	cgtctcctgtcgagtgacgaacc	*aatJ*-*lacZ* and *aatQ*-*lacZ*
JS50.f	gcatctagaccgccagtgtctgcaacc	*aatQMP* genes
JS51.r	gcagagctctcagtgcgggaggatcttgg	*aatQMP* genes
JS391K.f	aggaacttcaagatccccaattcgggaaatggtcaacgaagtgctcg	*aatQM* DnF-Gm
JS391K.r	tacaagaaagctgggtgatcttggcgaggaactgc	*aatQM* DnR-GWR
JS401K.f	tacaaaaaagcaggctggacttcagtggaatcgttcc	*aatQM* UpF-GWL
JS401K.r	tcagagcgcttttgaagctaattcgcgatgttcgacagcaacttgc	*aatQM* UpR-Gm
JS351.f	tacaaaaaagcaggctggaggagctggaggacatc	*aauR* UpF-GWL
JS351.r	tcagagcgcttttgaagctaattcggatgatccgttgcgccagg	*aauR* UpR-Gm
JS352.f	aggaacttcaagatccccaattcggaacacttcctccagcagtc	*aauR* DnF-Gm
JS352.r	tacaagaaagctgggtgcaggccgtacttcttcac	*aauR* DnR-GWR
JS100.f	tacaaaaaagcaggctccggatttcataagctggc	*aatJ* UpF-GWL
JS100.r	tcagagcgcttttgaagctaattcgggtaggagaagggaatcgaagc	*aatJ* UpR-Gm
JS101.f	aggaacttcaagatccccaattcggcgaggtcaacaagatctacg	*aatJ* DnF-Gm
JS101.r	tacaagaaagctgggtcctcggttcaatcggttgc	*aatJ* DnR-GWR
JS111.f	gcactcgaggccggggaaaaccgcgaaacaccc	*aatJ* gene
JS111.r	gcatctagattacatctgctcggcggccttgtcgg	*aatJ* gene
JS117.f	gcaggatccccggggaaaaccgcgaaacaccctgtccc	*aatJQMP* genes
JS117.r	gcatctagatcagtgcgggaggatcttggcgaggaactgc	*aatJQMP* genes
JS118.f	gcatctagagcatgtcttgctcggctgccagcaggc	*aauR* gene
JS118.r	gcagagctccccggcctattgcaggccgtacttcttcaccttgtcg	*aauR* gene

^a^Note that oligonucleotides used in the cloning of gene-deletion plasmids were designated as DnF-Gm, DnR-GWR, UpF-GWL or UpR-Gm in accordance with the original methods publication [43]

### Cloning of expression vectors for the *aatJQMP* and *aauR* genes

The *aauR*, *aatJ*, *aatQMP*, and *aatJQMP* genes were PCR-amplified from genomic DNA of *P. aeruginosa* PAO1 with the primers JS118.f-JS118.r, JS111.f-JS111.r, JS50.f-JS51.r, and JS117.f-JS117.r, respectively. The desired PCR products were gel purified and cloned into pCR-Blunt (Invitrogen) according to the manufacturer’s instructions. The *aauR*, *aatJ*, *aatQMP*, and *aatJQMP* genes were subsequently subcloned into the *Xba*I-*Sac*I, *Xho*I-*Xba*I, *Xba*I-*Sac*I, and *Kpn*I-*Xba*I sites of pBBR1MCS-5 [42] to yield the plasmids pJMS03, pJMS21, pJMS05, and pJMS19, respectively.

### Cloning of *aatJ*-*lacZ* and *aatQ*-*lacZ* reporters

The 5′ regulatory regions of the *aatJ* and *aatQ* genes were PCR-amplified from genomic DNA of *P. aeruginosa* PAO1 with the primers JS35P.f-JS43.r and JS35P.f-JS05.r, respectively, while the *lacZ* ORF was PCR-amplified from genomic DNA of *E. coli* MG1655 with the primers JS42.f-BL342.r (*aatJ* overlap) or JS06.f-BL342.r (*aatQ* overlap). The PCR-amplified 5′ regulatory regions of *aatJ* and *aatQ* were then fused to their cognate *E. coli lacZ* ORFs via fusion PCR [13], and the resulting *aatJ*-*lacZ* and *aatQ*-*lacZ* fusions were gel purified and subsequently cloned into pCR-Blunt (Invitrogen) to yield pJMS07 and pJMS08, respectively. The *aatJ*-*lacZ* and *aatQ*-*lacZ* fusions were then excised from pJMS07 and pJMS08 through double digestion with *Xba*I-*Spe*I, and the liberated fragments were separately cloned into the *Xba*I site of the promoter-less plasmid ΔP*lac*-pBBR1MCS-5 [13] to give pJMS09 and pJMS10, respectively.

### Construction of gene-deletion mutants of *P. aeruginosa*

The *aauR*, *aatJ*, and *aatQM* genes were deleted from the genome of *P. aeruginosa* PAO1 using established methods [43]. Briefly, the chromosomal mutations *aauR*::FRT-*aacC1*-FRT, *aatJ*::FRT-*aacC1*-FRT, and *aatQM*::FRT-*aacC1*-FRT were generated by electroporating *P. aeruginosa* PAO1 with the gene-deletion plasmids pJMS299, pJMS301, and pJMS303, respectively. Following selection on LB-agar supplemented with 30 μg mL^− 1^ gentamicin and verification of double-crossover mutations through PCR, the gentamicin-selection markers (*aacC1*) were removed via FLP-mediated excision to yield the unmarked Δ*aauR*, Δ*aatJ*, and Δ*aatQM* mutants. The Δ*aauR* mutants of *P. aeruginosa* PA14 and PAK were constructed in similar fashion.

### Cell-culture experiments

Analysis was performed in triplicate for each strain and/or condition. Individual colonies of wild-type *P. aeruginosa* and its isogenic mutants were each inoculated into 2.0 mL of LB (in a 16 × 125 mm culture tube) and subsequently grown at 37 °C at 200 rpm for 18 h. The LB-grown cells were collected via centrifugation and then washed two times with equal volumes of carbon- and nitrogen-free minimal media. The wash steps ensured the removal of residual carbon and nitrogen sources. Each washed cell pellet was ultimately suspended in an equal volume of carbon- and nitrogen-free minimal media, and the resulting cell suspensions were used at 1% (vol/vol), i.e., 2.0 μL, for the inoculation of 0.2 mL of test medium housed in a 96-well flat-bottom polystyrene plate (Falcon). Test media used for nitrogen-source analysis consisted of minimal media supplemented with 20 mM glucose and 10 mM NH_4_Cl_,_ L-glutamate, L-aspartate, L-glutamine or L-asparagine. Test media used for carbon-source analysis consisted of minimal media supplemented with 10 mM NH_4_Cl and 20 mM L-glutamate, L-aspartate, L-glutamine or L-asparagine. Inoculated plates were incubated in a BioTek Synergy™ H4 microplate reader set at 37 °C with medium shaking for 18 h. The absorbances or optical densities at 600 nm (OD_600_) were measured at 1.0 h intervals.

The Δ*aauR* mutant of *P. aeruginosa* PAO1 was electroporated with expression plasmids for *aauR* (pJMS03), *aatJ* (pJMS21), *aatQMP* (pJMS05) and *aatJQMP* (pJMS19). For controls, both wild-type *P. aeruginosa* PAO1 and the Δ*aauR* were transformed with empty pBBR1MCS-5. The resulting recombinant strains were grown in minimal media supplemented with 10 mM NH_4_Cl and either 20 mM L-glutamate or L-glutamine. Experiments were done exactly as described above except that growth media was supplemented with 30 μg mL^− 1^ gentamicin for plasmid selection.

### HPLC analysis of extracellular amino acids

Analysis was performed in triplicate for each strain and/or condition. Wild-type *P. aeruginosa* PAO1 and the ∆*aauR* mutant were grown in 10 mL of minimal media (in a 50 mL conical centrifuge tube) supplemented with 20 mM glucose and 20 mM L-glutamate, L-glutamine, L-aspartate or L-asparagine. Cultures were grown at 37 °C and 200 rpm. At 6.0 and 9.0 h post inoculation, 0.5 mL of culture was withdrawn, and cells were removed by centrifugation and subsequent passage of the supernatant through a 0.2 μm syringe filter. The resulting cell-free samples were then treated with *o*-phthalaldehyde to derivative the amino acids, which were subsequently separated through a reverse-phase Agilent Zorbax Eclipse amino acid analysis column (75 mm by 4.6-mm ID, 3.5-μm particle size) maintained at a temperature of 40 °C [44]. Separation involved the mobile phases A (40 mM Na_2_HPO_3_, pH 7.8) and B (45% acetonitrile:45% methanol:10% H_2_O) in a gradient program consisting of 2.0 min of 100% phase A, a 12.0 min linear gradient to 50% phase B, a 10 min linear gradient back to 100% phase A, and 4.0 min of 100% phase A at a flow rate of 1 mL min^− 1^ [44]. The dynamic range for the detection and quantification of derivatized amino acids was 45–450 pmol.

### LacZ assays

Analysis was performed in triplicate for each strain and/or condition. Wild-type *P. aeruginosa* PAO1, the ∆*aauR* and the *rpoN*::Ω-Km mutants were each electroporated with the *aatJ*-*lacZ* (pJMS09) and *aatQ*-*lacZ* (pJMS10) reporter plasmids. Following selection on LB agar supplemented with 30 μg mL-1 gentamicin, individual colonies were directly inoculated into minimal media supplemented with 20 mM L-alanine, 10 mM NH_4_Cl and 30 μg mL^− 1^ gentamicin. Bacteria were grown at 37 °C to an OD_600_ of ~ 0.2 and subsequently challenged with the addition of a final concentration of 20 mM of substrate: L-alanine, succinate, α-ketoglutarate, L-arginine, L-histidine, L-glutamate, L-aspartate, L-glutamine and L-asparagine. LacZ activity was measured at 1.0 and 3.0 h post addition of substrate using a microplate reader protocol as previously described [45]. Briefly, in a 96-well flat-bottom polystyrene plate (Falcon), 5.0 μL of cell culture was mixed with 45 μL of permeabilization solution (100 mM Na_2_HPO_4_, 80 μg mL^− 1^ CTAB, 40 μg mL^− 1^ deoxycholate, 2.0 mM MgSO_4_, 5.4 μL mL^− 1^ β-mercaptoethanol). The mixture was incubated for 30 min at 30 °C. Subsequently, 225 μL of substrate solution (100 mM Na phosphate, pH 7.0, 20 mM KCl, 1.0 mg mL^− 1^ *o*-nitrophenyl-β-D-galactoside, 2.7 μL mL^− 1^ β-mercaptoethanol) was added to the permeabilized-cell mixture, and the resulting reaction was incubated at 30 °C with shaking. The absorbance at 420 nm was measured every 1.5 min. Analysis of variance (ANOVA) was done using Dunnett’s post hoc test (α value, 0.05) to identify significant differences (*P* < 0.0001) in LacZ activities.

## Data Availability

All data generated or analyzed during this study are included in the submitted manuscript.

## Abbreviations

ABC: ATP-binding cassette
EBP: Enhancer-binding protein
TCS: Two-component signal transduction system
ND: Not detected.
